# Complete nucleotide sequence of strawberry vein banding virus Chinese isolate and infectivity of its full-length DNA clone

**DOI:** 10.1186/s12985-016-0624-1

**Published:** 2016-10-06

**Authors:** Mingfeng Feng, Hanping Zhang, Yuan Pan, Yahui Hu, Jing Chen, Dengpan Zuo, Tong Jiang

**Affiliations:** School of Plant Protection, Anhui Agricultural University, Hefei, 230036 People’s Republic of China

**Keywords:** Strawberry, Strawberry vein banding virus, Complete genome sequence, Vacuum infiltration, Infectious clone

## Abstract

**Background:**

Strawberry vein banding virus (SVBV) is a double-stranded DNA plant virus, which has been found in North America, Australia, Brazil, Japan, Europe and several provinces of China. Infected strawberry plants exhibit mild vein-banding symptoms and chlorosis along the veins. It is one of the most economically important diseases in Asiatic, European and North American strawberry-growing areas.

**Findings:**

The complete genome of an SVBV Chinese isolate (SVBV-CN) was isolated and cloned from a naturally infected strawberry (*Fragaria × ananassa* cv. Sachinoka) sample found in Shenyang city of Liaoning province. Sequence analysis revealed a complete genome of 7864 nucleotides (nts) that indicated SVBV-CN was most closely related to SVBV from the United States (SVBV-US) with a sequence similarity of 85.8 %. Two major clades were identified based on phylogenetic analysis of the complete genome sequences of caulimoviruses. SVBV-CN clustered together with SVBV-US, whereas other caulimoviruses formed a separate branch. Agrobacterium-mediated inoculation of *Fragaria vesca* with an infectious clone of SVBV-CN results in systemic infection with distinct symptoms of yellowing bands along the main leaf veins. This suggests that the SVBV-CN infectious clone can recapitulate the symptoms observed in naturally infected strawberries, and therefore is likely the causal agent of the original disease observed in strawberries. Furthermore, strawberry plants inoculated with the infectious clone using vacuum infiltration developed symptoms with a very high infection rate of 86–100 % in 4-5 weeks post-inoculation. This compares to an infection rate of 20–40 % in 8–9 weeks post-inoculation using syringe-inoculation.

**Conclusions:**

The complete nucleotide sequence of SVBV from a naturally infected strawberry was determined. Agroinfiltration of strawberry plants using an infectious clone of SVBV-CN resulted in symptoms typically found in infected strawberries from Shenyang city of Liaoning province in China. This is the first report describing an infectious clone of SVBV-CN, and that vacuum infiltration can be potentially used as a new and highly efficient means for inoculation of strawberry plants.

**Electronic supplementary material:**

The online version of this article (doi:10.1186/s12985-016-0624-1) contains supplementary material, which is available to authorized users.

## Background

The first report of strawberry vein banding virus (SVBV) was in 1955, which has been followed by additional research in Europe and America [1]. The virus was shown to be transmissible by grafting or by several aphid species in a semi-persistent manner [2], and distributed world-wide on cultivated strawberries [3]. SVBV could only be detected on *Fragaria* species in naturally occurring infections [4]. Typical symptoms observed in infected indicator plants, *F. vesca* or *F. virginiana*, are bands of yellowing along the main leaf veins and partial reduction in growth potential and twisting of leaflets [4]. However, cultivated strawberry plants are symptomless when infected with SVBV alone [5], while co-infection of SVBV with other strawberry viruses, strawberry mottle virus and strawberry mild yellow edge virus, has the potential to cause serious symptoms in strawberry plants [6].

SVBV is a plant virus characterized by the equiaxed shape and 40 to 45 nm particles [7]. The virus contains a double-stranded (ds) DNA genome and is classified in the genus *Caulimovirus* of the family *Caulimoviridae* [8]. So far, the only complete nucleotide sequence of SVBV is for an isolate from the United States (accession number: X97304) [3]. SVBV-US has a 7.8 kb circular dsDNA genome with one single-stranded discontinuity on each DNA strand, with seven open reading frames with the potential to code for seven proteins [3]. SVBV-infected cells contain cytoplasmic inclusion bodies, typical of those produced by other caulimoviruses [7]*.*

SVBV has been found frequently associated with most sources of strawberry virus disease investigated, but the role of the virus in the aetiology of the disease remains to be determined [9]. The natural host range of SVBV is limited to strawberry, and no alternative host for SVBV has been identified to facilitate biological studies [10]. To overcome these difficulties, an infectious clone of SVBV is essential for the study of this virus. An infectious clone was generated and used to inoculate strawberry plants, which allowed us to investigate the ability of SVBV to replicate, move cell-cell, spread systemically and assemble into virus particles [11]. Here, we have determined the complete nucleotide sequencing of an isolate of SVBV from China and successfully generated an infectious clone of SVBV-CN.

## Results and discussion

### Genome organization of SVBV-CN

The complete nucleotide sequence for the SVBV-CN genome was determined, and shown to consist of 7864 nts (GenBank accession number KP311681). The overall genomic organization of SVBV-CN has extensive similarities to that of other caulimoviruses, especially CaMV, including seven putative open reading frames (ORFs), a large untranslated region and some small intergenic regions between ORFs. Computer analysis revealed that the SVBV genome has the potential to code for seven proteins of calculated weight 37.9, 18.5, 13.5, 55.4, 80.8, 59.8 and 12.4 kDa.

By comparison to CaMV the protein encoded by ORF I (position 70-1059) is believed to function in cell-to-cell movement; ORF II (position 1062-1550) produces a putative aphid transmission protein; ORF III (position 1551-1904) encodes a non-sequence specific DNA-binding protein [3]; ORF IV (position 1907 to 3322) encodes a putative coat protein; ORF V (position 3414-5528) encodes a polyprotein precursor that contains three conserved domains, peptidase, reverse transcriptase (RT) and ribonuclease H (RNase H) (http://www.ncbi.nlm.nih.gov/cdd) [12]; ORF VI (position 5540-7102) encodes 59.8 kDa protein that is the main component of viral inclusion bodies or viroplasms [13]. This protein is similar to the CaMV transactivator protein and probably controls virus-host specificity [14]. Whether ORF VII (position 7618-77) encodes a protein is still not clear, and there is no reliable evidence for its presence in strawberry plants infected with SVBV [3].

The complete genome sequence of SVBV-CN contains some long and short untranslated regions between adjoining ORFs. A long intergenic region of 515 nts is present between ORF VI and ORF VII, and there are two short regions located upstream (91 nts) and downstream (11 nts) of ORF V, respectively. Additionally, there is a two-nucleotide intergenic space between ORF I and ORF II, and between ORF III and ORF IV. No intergenic region is found between ORF II and ORF III, which are two continous ORFs.

Sequence comparison showed that the overall nucleotide sequence similarity between SVBV-CN and SVBV-US was 85.8 %, whereas SVBV-CN shared only 43.3–44.8 % nucleotide sequence similarity with other reported caulimoviruses (Table 1). Furthermore, alignment of the amino acid sequence of SVBV-CN ORFs with those of other caulimoviruses, revealed very low similarities (3.8–32.8 %). Only ORF V of SVBV-CN had a relatively high amino acid sequence similarity (49.0–52.7 %) with those of other caulimoviruses (Table 1). This suggests that ORF V of SVBV-CN has a closer evolutionary relationship with the caulimoviruses.

**Table 1:** Nucleotide and amino acid sequence similarities (%) among SVBV-CN and SVBV-US and other caulimoviruses

Virus name	Accession number	Genome^a^	IR^a^	I^b^	II^b^	III^b^	IV^b^	V^b^	VI^b^	VII^b^
SVBV-US	X97304	85.8	85.1	86.6	69.6	80.2	90.4	94.0	86.9	72.1
CaMV-CM1841	V00140	44.8	28.0	32.1	16.1	16.8	29.3	52.4	19.6	16.1
CaMV-Cabb	KJ716236	44.5	28.1	31.4	17.3	16.8	29.6	52.5	19.6	17.2
CaMV-IRN20	AB863155	44.6	27.8	31.4	16.8	16.8	29.3	52.7	19.8	14.0
CaMV-XJ	AF140604	44.8	28.4	31.3	16.0	16.8	28.8	52.8	19.8	15.1
CaMV-JPNUV26	AB863165	44.8	28.0	31.7	16.1	16.8	29.8	52.3	19.6	16.1
CERV-Indian	AJ853858	43.6	33.1	29.4	16.7	14.2	27.7	51.1	18.8	15.2
CERV-Hungary	X04658	43.7	33.7	29.4	16.8	14.2	28.0	51.3	19.1	15.2
FMV	X06166	44.1	29.9	31.8	14.0	23.8	30.4	51.9	18.9	6.0
DaMV	JX272320	44.7	42.0	32.4	17.2	16.1	25.3	49.0	19.7	7.9
HrLV	JX429923	43.3	28.0	30.8	16.7	16.2	28.4	50.7	20.4	3.8
MiMV	AF454635	43.4	38.9	32.8	16.1	14.3	25.5	52.5	22.1	9.2
SPuV	JQ926983	44.7	32.3	30.4	25.6	23.1	27.5	51.9	18.2	NA
LLDAV	EU554423	44.0	28.5	29.9	16.5	17.1	27.8	49.6	18.1	NA

^a^Nucleotide sequence similarity

^b^Amino acid similarity

*NA* Not assessed

### Phylogenetic analysis of SVBV-CN and affinities to other caulimoviruses

**Fig. 1:**
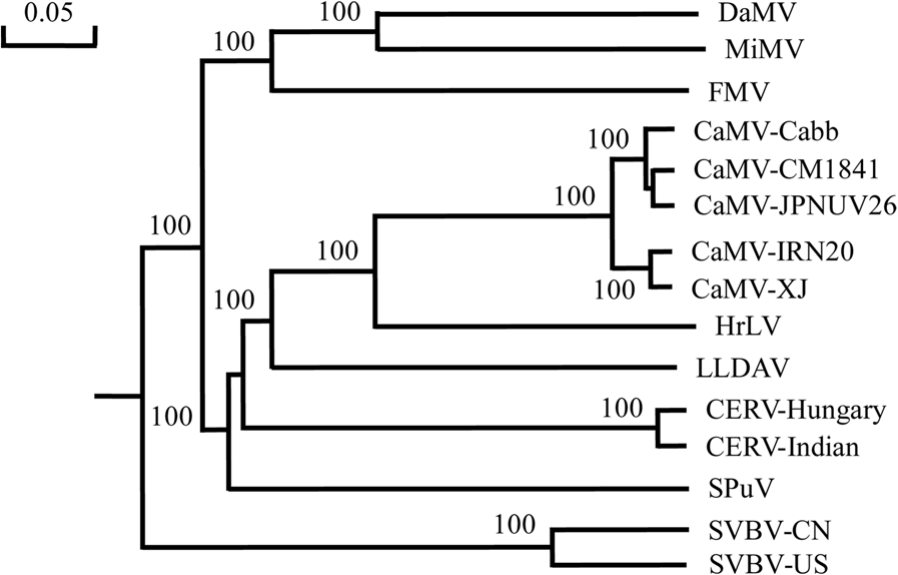
Phylogenetic tree based on complete genome sequences of selected caulimoviruses. The tree was constructed by the observed-divergency method. Branch significance was evaluated by constructing 1000 trees in bootstrap analysis, and the bootstrap values (>90 %) are shown. Abbreviations and GenBank accession numbers are as follows: SVBV China isolate (SVBV-CN, KP311681), SVBV United States isolate (SVBV-US, X97304), cauliflower mosaic virus CM1841 isolate (CaMV-CM1841, V00140), CaMV Cabb isolate (CaMV-Cabb, KJ716236), CaMV-IRN20 isolate (CaMV-IRN20, AB863155), CaMV Xingjiang isolate (CaMV-XJ, AF140604), CaMV-JPNUV26 isolate (CaMV-JPNUV26, AB863165), carnation etched ring virus Hungary isolate (CERV-Hungary, X04658), CERV Indian isolate (CERV-Indian, AJ853858), dahlia mosaic virus (DaMV, JX272320), figwort mosaic virus (FMV, X06166), horseradish latent virus (HrLV, JX429923), mirabilis mosaic virus (MiMV, AF454635), soybean putnam virus (SPuV, JQ926983), lamuim leaf distortion-associated virus (LLDAV, EU554423)

In order to characterize the relationship between SVBV-CN and other caulimoviruses, a phylogenetic dendrogram based on the complete nucleotide sequence of SVBV-CN and 14 other caulimoviruses sequences, was constructed using the DNAMAN Version 8 (Lynnon Corporation, Canada) software with 1000 bootstrap trials. Phylogenetic analysis resulted in two major clades. SVBV-CN clustered together with the SVBV-US isolate, whereas other caulimoviruses formed a separate branch. Furthermore, several sub-branches were identified within the caulimoviruses (Fig. 1).

### Infectivity of SVBV-CN in *F. vesca* and *Nicotiana* plants

**Fig. 2:**
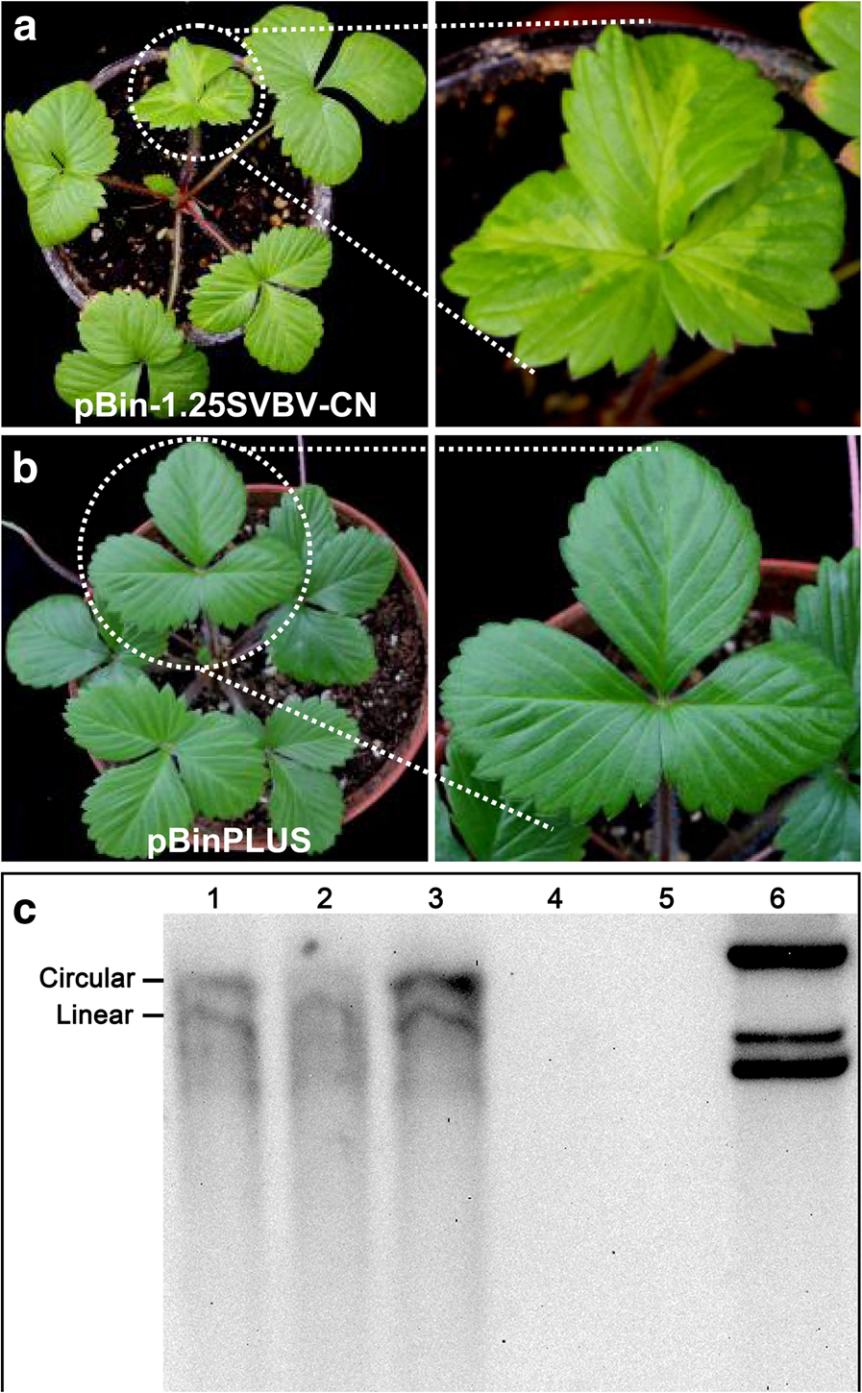
Symptoms caused by SVBV-CN on strawberry (*F. vesca*) plants and Southern blot analysis of SVBV-CN DNA. **a:** Yellow vein banding is observable around the mid vein of leaves of strawberry (*F. vesca*) plants inoculated with SVBV-CN; **b**: Symptomless strawberry (*F. vesca*) plants inoculated with empty vector pBINPLUS*.* Panels on the right are magnified images of leaves from the panels on the left. **c:** Southern blot of DNA isolated from plants inoculated with SVBV-CN (Lanes 1-3) or empty vector (Lanes 4-5). Lane 6 contains pUC-1.0SVBV-CN plasmid DNA. DNA was detected using a digoxigenin-labeled probe specific for SVBV-CN DNA. The positions of circular and linear forms of the viral DNA are indicated on the left side

To investigate the biological role of SVBV-CN, the ability of the SVBV-CN full-length clone to infect plants was assessed by *agrobacterium*-mediated inoculation to *F. vesca* and *Nicotiana* plants. *F. vesca* plants inoculated with cloned SVBV-CN DNA developed bands of yellowing along the main leaf veins within 30 days of inoculation (Fig. 2a). These symptoms are typical for SVBV-US infection in *F. vesca* [15]*.* In contrast, *F. vesca* plants inoculated with the empty vector (pBinPLUS) did not develop any symptom even 3 months post-inoculation (Fig. 2b). However, the inability of cloned SVBV-CN DNA to cause disease symptoms in *N. benthamiana, N. tabacum, N. glutinosa* or *N. tabacum* var. *Samsun* (NN) (data not shown) suggests a narrow host-range similar to that observed in naturally occurring infections [4]. These data strongly suggest that SVBV-CN sequences contained within pBin-1.25SVBV-CN are infectious and able to confer disease symptoms similar to those observed for SVBV-US. To confirm infectivity, Southern blot analysis was conducted. *F. vesca* plants agroinoculated with pBin-1.25SVBV-CN contained SVBV-CN DNA (Fig. 2c, lanes 1, 2 and 3), whereas no viral DNA could be detected in plants agroinoculated with pBinPLUS (Fig. 2c, lanes 4 and 5). Taken together, these results indicate that we have successfully cloned full-length genomic DNA of SVBV-CN and that the DNA is able to cause disease.

### Comparison of infectivity rates of SVBV-CN in *F. vesca* using different inoculation methods

To determine the efficiency of infection for different inoculation methods, we performed syringe inoculation and vacuum infiltration as a means to infect *F. vesca* plants. As shown (Table 2), symptoms typical of an SVBV infection developed in 20–40 % of plants inoculated by syringe inoculation approximately 2 months post-inoculation. Syringe inoculation has been used previously for SVBV, resulting in 100 % infection in strawberry plants [10]. However, in our hands we only achieved a maximum infection rate of 40 %. We attribute this to the thinner leaves and denser leaf tissue, which made syringe inoculation very difficult. Therefore, we explored a novel modified inoculation procedure to infect strawberry by vacuum infiltration. *F. vesca* plants inoculated using vacuum infiltration developed symptoms at a very high infection rate of 86–100 % (Table 2). In addition, the vein banding symptoms developed in vacuum-infiltrated plants 4–5 weeks post-inoculation, as compared to 8–9 weeks for syringe infiltration. This indicates that vacuum infiltration of SVBV-CN is very efficient and possibly enhances the development of SVBV symptoms. Our conclusion is that vacuum infiltration could potentially be used as a new method for the delivery of infectious clones of SVBV, and possibly other viruses, to plants*,* especially those that prove difficult to inoculate by syringe infiltration, as observed for *Fragaria* plants*.*

**Table 2:** Infection rate for two different methods for inoculation of strawberry (*F. vesca*) plants with SVBV-CN

Replicate	Syringe infiltration		Vacuum infiltration	
	No. of plants infected/inoculated	% Infection rate	No. of plants infected/inoculated	% Infection rate
I	3/15	20	12/14	86
Control	0/5	0	0/5	0
II	5/15	33	13/13	100
Control	0/5	0	0/5	0
III	6/15	40	14/15	93
Control	0/5	0	0/5	0

The above results demonstrate the infectivity of cloned SVBV-CN DNA, which causes symptoms typical of the disease observed in strawberry, thus fulfilling Koch’s postulates. This suggests that SVBV-CN is the causal agent of the disease symptoms observed in infected strawberry in Liaoning province in China. To our knowledge, this is the first report describing generation of an infectious clone of SVBV DNA in China. Along with development of an efficient inoculation method for strawberry plants using vacuum infiltration, this will allow us to further examine the biological properties of SVBV-CN and to possibly develop a novel viral vector for gene transient expression and/or virus-induced gene silencing.

## Materials and methods

### Virus sources and DNA extraction

SVBV-CN obtained from a naturally infected strawberry (*Fragaria × ananassa* cv. Sachinoka) sample found in Shenyang city of Liaoning province was kindly provided by Prof. Zhihong Zhang at Shenyang Agricultural University. The sample showed symptomless on leaves but growth potential attenuated. Total DNA was extracted from strawberry leaves using CTAB method as described [16].

### Sequencing of complete nucleotide sequence

Based on complete nucleotide sequences of SVBV-US and other calimoviruses in GenBank (accession number X97304, V00140, KJ716236, AB863155, AF140604, AB863165, X04658, JX272320, X06166, JX429923, AF454635, JQ926983 and EU554423), three degenerate primer pairs (Frag1F/Frag1R, Frag2F/Frag2R and Frag3F/Frag3R; Additional file 1: Table S1) were designed for amplifying three overlapping segments corresponding to the complete genome of SVBV-CN. PCR was performed in 50 μL reactions using 1 μL DNA template extracted from infected plants, 1 μM of each gene-specific primer, 2 units of Q5® High-Fidelity DNA Polymerase (New England Biolabs Inc., USA), and buffer provided by the manufacturer (containing 1.5 mM MgCl_2_). The PCR reaction was conducted as follows: 94 °C for 5 min, 30 cycles at 94 °C for 30 s, 55 °C for 30 s, and 72 °C for 40 s, with a final extension at 72 °C for 10 min. The PCR products with a 3′-A addition were inserted into pUC-T Vector (CWBIO, Beijing, China) using TA-cloning strategy followed by transformation of chemically competent *Escherichia coli* DH5α cells [17]. Putative clones were sequenced to confirm successful cloning. To generate the complete genome sequence of SVBV-CN, the sequence from each of the three segments were assembled and analyzed with the aid of SeqMan software (Lasergene 7.1.0, DNASTAR Inc., USA). The three overlapping segments were assembled into a full-length genome using specific restriction endonucleases or homologous recombination, and the complete genome inserted into pUC vector (CWBIO, Beijing, China) to produce pUC-1.0SVBV-CN, containing one copy of the full-length virus genome.

### Phylogenetic analysis

Sequence similarity searches were performed using the BLAST program (http://www.ncbi.nlm.nih.gov/). SnapGene Viewer (GSL Biotech, Chicago, IL) was used to search for potential ORFs in the genome. Conserved domains in the genomic sequences were identified in Conserved Domain Search (CD-Search) in NCBI (http://www.ncbi.nlm.nih.gov/cdd) [12]. Phylogenetic trees were constructed using the full optimal alignment and neighbor-joining method options with 1000 bootstrap replications available in DNAMAN Version 8 (Lynnon Corporation, Canada) software. Nucleotide sequence data used in this study were obtained from the GenBank database: SVBV United States isolate (SVBV-US, X97304), cauliflower mosaic virus CM1841 isolate (CaMV-CM1841, V00140), CaMV Cabb isolate (CaMV-Cabb, KJ716236), CaMV IRN20 isolate (CaMV-IRN20, AB863155), CaMV Xingjiang isolate (CaMV-XJ, AF140604), CaMV JPNUV26 isolate (CaMV-JPNUV26, AB863165), carnation etched ring virus Hungary isolate (CERV-Hungary, X04658), CERV Indian isolate (CERV-Indian, AJ853858), dahlia mosaic virus (DaMV, JX272320), figwort mosaic virus (FMV, X06166), horseradish latent virus (HrLV, JX429923), mirabilis mosaic virus (MiMV, AF454635), soybean putnam virus (SPuV, JQ926983), lamuim leaf distortion-associated virus (LLDAV, EU554423).

### Construction of infectious clone of SVBV

Cloned DNA containing 0.72 and 0.53 copies of full-length virus genomic DNA was generated by PCR using primers SY1F/SY1R or SY2F/SY2R (Additional file 1: Table S1: enzyme sites underlined) and pUC-1.0SVBV-CN as template. The resulting products were restricted with *Sal* I*/Kpn* I and *Kpn* I/*Sma* I and cloned into similarly digested pUC to produce pUC-0.72SVBV-CN and pUC-0.53SVBV-CN respectively. Plasmid pUC-0.53SVBV-CN was digested with *Kpn* I and *Sma* I and the resulting 4.3 kb fragment introduced into the binary vector pBinPLUS to produce pBin-0.53SVBV-CN [18]. Plasmid pUC-0.72SVBV-CN was restricted with *Sal* I and *Kpn* I and the 0.72 kbp fragment of SVBV-CN inserted into similarly digested pBin-0.53SVBV-CN to generate pBin-1.25SVBV-CN, containing 1.25 copies of the full-length SVBV-CN genome.

### Agroinoculation of plants

Plasmid pBin-1.25SVBV-CN and the binary vector pBinPLUS were transformed into *Agrobacterium tumefaciens* strain EHA105 by electrotransformation. Transconjugants were selected on kanamycin (50 μg/mL) and streptomycin (50 μg/mL). *A. tumefaciens* cultures were grown at 28 °C for 48 h, after which a fine syringe was used to inoculate into the leaves of *F. vesca, Nicotiana benthamiana, N. tabacum, N. glutinosa and N. tabacum* var. *Samsun* (NN) at the 5-6 leaf stage. In addition, *F. vesca* plants were also inoculated using vacuum infiltration as follows. Whole strawberry seedlings were immersed in the *A. tumefaciens* inoculum in a 250 mL beaker, and placed in a vacuum chamber at 101 KPa atmospheric pressure for 30 s. After vacuum infiltration, the strawberry plants were rinsed with distilled water and then transplanted into pots containing a mixture of 1:1 (v/v) peat : vermiculite. Inoculated plants were grown at 25 °C in an insect-free chamber with a 16 h photoperiod and 75 % relative humidity.

### Hybridization analysis

Mock-inoculated and SVBV-infected strawberry leaves were harvested at 40 dpi. Total DNA was extracted from strawberry leaves using the CTAB method and fractionated by 1 % agarose gel electrophoresis in TBE buffer (90 mM Tris-borate, 2 mM EDTA, pH 8.3), and then transfer to Hybond-N^+^ membranes (Amersham Pharmacia, Little Chalfont, Buckinghamshire, England). After alkali denaturation and neutralization, hybridization was detected with digoxigenin-labeled probes specific for SVBV using a DIG High prime DNA labeling and detection starter kit II (Roche) according to the manufacturer’s instructions.

## Additional file

Additional file 1: Table S1. Sequence of primers used for PCR amplification and cloning. (DOC 22 kb)
