# Supplementary material for: Complete nucleotide sequence of strawberry vein banding virus Chinese isolate and infectivity of its full-length DNA clone
Source: Virol J. 2016 Oct 6;13:164. doi: 10.1186/s12985-016-0624-1 (PMC5052798; doi:10.1186/s12985-016-0624-1)
Supplement: Additional file 1: Table S1. — Sequence of primers used for PCR amplification and cloning. (DOC 22 kb) [file 12985_2016_624_MOESM1_ESM.doc]

**Table S1** Sequence of primers used for PCR amplification and cloning.

| Primer | Nucleotide sequence (5′-3′) |
| --- | --- |
| Frag1F | TACTCGAGCGCAAGCTGAGAAG |
| Frag1R | TCATGCCACCGAAGACTGCGTCTC |
| Frag2F* | GGTACCGACAGGTAATTATTGGTATCCTAC |
| Frag2R* | TACATGCTTGTTTGCTGTACACATAC |
| Frag3F | CAGAATCGTCATCAGATGAATCAGACGAC |
| Frag3R* | GGTACCTTTAATTATGGCTTGTTGATGGCCTC |
| SYF1* | GTCGACGTAATCAGACTACCAAAGTG |
| SYR1* | GGTACCCATACTCGCACCTGTATCAAC |
| SYF2* | GGTACCTGTACAGCAAACAAGCATG |
| SYR2* | CCCGGGTCTCGCGGTTGTCATTCTC |

*-Underlined sequences manifest recognition site of restriction endonuclease.
